# Prevalence of Overweight, Obesity, and Thinness in Cameroon Urban Children and Adolescents

**DOI:** 10.1155/2013/737592

**Published:** 2013-06-19

**Authors:** Ponce Cedric Fouejeu Wamba, Julius Enyong Oben, Katherine Cianflone

**Affiliations:** ^1^Laboratory of Nutrition and Nutritional Biochemistry, Department of Biochemistry, Faculty of Science, University of Yaoundé 1, P.O. Box 8418, Yaoundé, Cameroon; ^2^Centre de Recherche Institut Universitaire Cardiologie & Pneumologie de Québec, Y4323, 2725 Chemin Sainte-Foy, Québec, QC, Canada G1V 4G5

## Abstract

*Objective*. This study examined the prevalence of thinness, overweight, and obesity in Cameroon children ranging from 8 to 15 years old using several published references as evaluation tools. *Methods*. A stratified sample was used with eleven schools randomly selected, and data from 2689 children (52.2% girls) ranging from 8 to 15 years were analyzed. Weight and height were recorded and BMI was calculated. BMI cutoffs used to define nutritional status grades included two international and three national published indices which were compared to our database-derived cutoffs. *Results*. A prevalence of 9.5% thinness and 12.4% overweight including 1.9% obesity according to international references was detected. A 2.2% low-weight-for-age, 5.7% low-height-for-age, and 5.2% low-weight-for-height were identified. Overall, there were significant differences using calculations based on our database versus published reference values and between boys versus girls. *Conclusions*. This study demonstrates that prevalence of thinness, overweight, and obesity is similar to that of other leading-emerging countries reported within the last decade, yet it is still lower than prevalence in developed countries. Ethnic background and social environment have impact on prevalences, highlighting the importance of evaluating the Cameroon population based on locally derived database.

## 1. Introduction

Obesity has become a global health problem. According to the World Health Organization (WHO) in 2005 about 1.6 billion adults were affected worldwide, with about 400 million adults categorized as obese [1]. In the Cameroon adult population, both overweight (21.6% men and 28.6% women) and obesity (6.5% men and 19.5% women) are prevalent and are increasing in both rural and urban areas [2–4]. The higher sociodemographic levels, evaluated based on education level, demonstrated a 2-to-3.5-fold risk of being overweight or obese in men [5]. This data, along with others, suggests the ongoing development of an obesogenic environment in Cameroon, as is seen in many developing countries undergoing rapid urbanization and social changes [4, 5].

In developed and developing countries, with the reduction of underweight status, there has been widespread concern over the increase in overweight and obesity in children [5, 6]. This results in the simultaneous occurrence of undernutrition and obesity at the childhood level in many developing countries [5]. Childhood obesity is considered to be a precursor of adverse health effects in adulthood, as overweight children are more likely to become overweight adolescents and adults; 2.8 times more likely in one study in Chinese children [7].

The definition of both overweight and obesity in children and adolescents is still a matter of debate [8–11]. To date, body mass index (BMI), calculated as weight (kg) per height (m^2^), can be easily assessed at low cost and is strongly associated with body fat and health risks in adults [12]. In children and adolescents, BMI has increasingly been accepted as a valid indirect measure of fat mass with age and gender specific cutoffs proposed in various studies; but there remains a wide array of references used, based on different populations, including weight-for-height (ideal weight for height or *z*-score), BMI percentile, skin fold thickness, and waist circumference [8, 9, 11, 13–15].

Recent studies in Cameroon specifically have pointed out the association of obesity with hypertension and diabetes in adults [2, 16]. However, there is a lack of data concerning overweight and obesity assessment in childhood and adolescence in Cameroon, although, clearly, the risk of childhood obesity leading to adult morbidity is of great public health significance. Hence, this cross-sectional study was designed to (i) estimate the prevalence of grades of nutritional status (under- and overweight) in urban zones in Cameroon and (ii) examine this prevalence with respect to existing references and internationally available data on childhood and adolescence.

## 2. Materials and Methods

### 2.1. Sample and Procedures

Subjects were recruited from a cross-sectional school-based survey: the Douala Child and Adolescent Obesity Study in Cameroon (DCAO study). A total of 2689 school children ranging from 8 to 15 years old were recruited between February and May 2010 in the city of Douala, including girls (*n* = 1404) and boys (*n* = 1285). A stratified sampling procedure was used in selecting schools and a quota sampling was used in each school. Data were widely collected so as to incorporate all social strata, as well as ethnic groups, including minorities. Data from subjects aged 8 to 15 years old were pooled for further analysis. This study was approved by the School Administration and the National Ethics Committee. Signed informed consent was obtained from parents or guardians.

### 2.2. Measures

Anthropometric variables were measured according to existing standards by trained enumerators. Height was measured without shoes to the nearest 0.1 cm using a portable stadiometer, and body weight was measured to the nearest 0.1 kg using an indoor weighing scale with the student's shoes, coats, and other heavy outerwear removed. Height and weight were used to calculate BMI as body mass (kg)/square of height (m^2^).

### 2.3. Choice of Reference Tools and Cutoffs for Grouping

Our database-derived reference (Database) was first built according to various methods and second compared to other published references for the assessment of grades of nutritional status based, respectively, on BMI, weight for height, weight for age, and height-for-age. The LMS method was used to summarize the dataset in three smooth age specific values of skewness (*L* or Lamda), median (*M* or Mu), and coefficient of variation (*S* or Sigma); then each age and gender-specific BMI cutoffs or percentiles were derived from our Database using the formula: *M*(1+*LS*_*z*_)^1/*T*^, where *z* indicates the *z* score for required cutoff, which corresponded to BMI values of 16, 17, 18.5, 25, and 30 kg/m² at 18 years (Method 1), 85th and 95th percentiles (Method 2) and 97th BMI percentile (Method 3). Our Database was then compared to different references using the same methodology.

**Method 1**

Database percentiles passing through BMI values of 16, 17, 18.5, 25, and 30 kg/m² at 18 years were compared to Cole 2007 and IOTF similar published data [8, 9, 17].

**Method 2**

Database-derived 85th and 95th BMI percentiles were compared with similar cutoffs as the old US (Must et al.) [18], the new US (CDC) [19, 20], and the WHO 2007 reference [21].

**Method 3**

The 97th centile from database was compared with the Europe-French 97th BMI percentile reference [11].

WHO 2007 reference [21] and CDC references [20] were used to assess the level of undernutrition including wasting, stunting, and underweight. Wasting was defined as weight below 2 standard deviations (SD) from the median for weight-for-height. Further, moderate wasting (wasting I) and severe wasting (wasting II) are defined, respectively, as below 2 SD and below 3 SD. Stunting was defined as height below 2 SD from the median for height-for-age. Underweight was defined as weight below 2 SD from the median for weight-for-age.

### 2.4. Statistics

Quantitative data are presented as median with coefficient of variation and skewness or means with standard error of the mean where stated. Differences between boys versus girls were tested by Student's *t*-test. Frequency data are given as percentages or ratios, frequency of overweight; obesity and thinness were standardized by age and gender. Comparison between genders and references used was performed using the Chi-square test. The significance level was set at *P* < 0.05. Data were analyzed with SPSS 10.0 for Windows (SPSS Inc., 1999) and GraphPad Prism 5.

## 3. Results

Table 1 outlines the principal age and gender specific characteristics of the database sample in boys (Table 1(a)) and girls (Table 1(b)). Little gender differences on anthropometric variables (weight, height, and BMI) were observed at age 8, 9, and 10 years, but all anthropometrics were significantly higher in girls versus boys especially at 11, 12, 13, and 14 years; all *P* < 0.01 (data not shown).

Based on the *LMS* (Lamda, Mu, and Sigma) method, mean (*M*), skewness (*L*), and coefficient of variation (*S*) used for the determination of different BMI cutoffs and *Z* scores were calculated from our Database as shown in Tables 2 and 3, for boys and girls, respectively. BMI values increase with age in both boys and girls, with higher values for girls at all ages.  Table 4 presents age-related BMI cut-off points for various degrees of thinness, overweight, and obesity between 8 and 15 years, designed by the *LMS* method to match adult cutoffs. Overall, cut-off values increase with age in both boys and girls; a marked shift towards lower values was observed at 11 years old for girls and 13 years old for boys, particularly with respect to thinness cut-offs, while overweight and obesity cutoffs were less affected.

**Figure 1:**
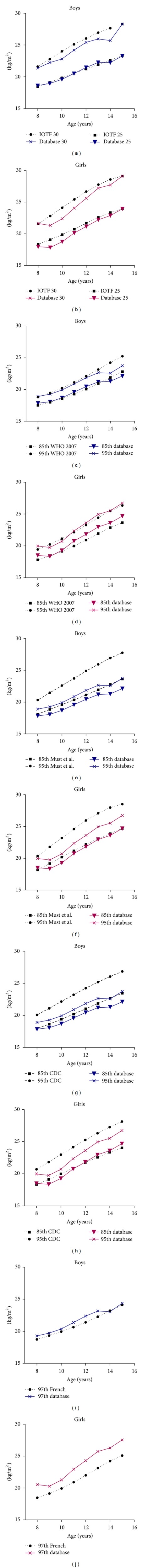
Comparison of age- and gender-specific body mass index (BMI) cut-off curves of our Database population of 8 to 15 years old presented relative to different published reference groups: International Obesity Task Force (IOTF) cutoffs for overweight and obesity matching, respectively, BMI of 25 and 30 kg/m^2^ at 18 years old (a and b), World Health Organization (WHO) 2007 85th and 95th percentiles (c and d), Must et al. or “old United States” 85th and 95th percentiles (e and f), Center of Disease Control (CDC) or “new United States” 85th and 95th percentiles (g and h), and Europe-French 97th percentile for overweight including obesity (i and j). In all panels, corresponding cutoffs derived from our Database are presented as solid lines presented beside reference cutoffs with dotted lines.

In Figure 1 ((a) to (j)), five different reference cut-offs are compared to our Database-derived cut-offs using the same calculation methodology. In panels ((a) and (b)), other than the cutoff for overweight boys, in general the IOTF reference [8] cut-offs were higher versus our Database-derived cut-offs. In panels ((c) and (d)), WHO 2007 [21] and 85th and 95th percentiles, defining, respectively, overweight and obesity, were closer to corresponding percentiles in our Database, especially at younger ages. In panels ((e) to (h)), US references, based on Must et al. [18] and CDC for 85th and 95th percentiles (overweight and obesity, resp.) [19], were all higher than our corresponding Database percentiles for both boys and girls. Of note, for girls, closer values for the 85th percentile were obtained in older versus younger girls while no such observation was made for boys, where the 95th percentile was shifted closer to the US 85th percentile. The French reference group [11] (panels (i) and (j)) defined as the 97th percentile was lower than our Database 97th percentile, with the difference more evident in girls versus boys.

Table 5 summarizes the prevalence of overweight excluding obesity and obesity in our Database, evaluated according to the age and gender definitions used in the different references. The prevalence of overweight ranges from 6.4% to 8.2% in boys and from 10.7% to 17.2% in girls, whereas prevalence of obesity ranges from 1.4% to 5.5% in boys and from 2.4 to 8.6% in girls. Regardless of the analysis method used, our Database-derived prevalence was significantly different from the corresponding references used (all *P* < 0.001).

Table 6 summarizes the prevalence of undernutrition in our study population. Prevalence of underweight and stunting according to WHO 2007 reference was higher in boys as compared to girls, 5.5% versus 4.8% and 9.2% versus 2.4%, respectively; while wasting was about 1.5 times higher in girls as compared to boys at 2.9% versus 1.6%. Thinness, defined by BMI cut-offs (Cole 2007 and our Database), yields about the same prevalence in boys and girls, 9.4% versus 9.7% and 5.5% versus 4.9%, respectively, using Cole 2007 and our Database cutoffs.

## 4. Discussion

This study was conducted in 2010 in urban Cameroonian children and adolescents aged 8 to 15 years old. According to Cole et al. 2007 and IOTF references, we report an overall prevalence of 9.5% thinness and 12.4% overweight including 1.9% obesity. Prevalence of thinness was comparable to that of other developing regions [5, 22], while prevalence of overweight and obesity was lower than that of reported data from Europe and US [23]. The overweight prevalence comes close to the bottom end of the bracket observed in Europe, which is unexpectedly high for this country. These results then support the previously reported coexistence of both thinness and overweight in developing countries, with the increasing rate of obesity overtaking that of thinness [5, 22, 24].

This study has several limitations that need to be considered. First the collection of data was only in one urban area of one region of the country, which limits the potential conclusions to that area, and is not applicable to general rural areas. From a total of 3239 children and adolescents selected, 550 (16.9%) were eliminated because of missing data or they were not within the proposed age range (below 8 or above 15 years old). Nonetheless, the final sample within inclusion criteria included 2689 subjects comprising 47.8% boys (*n* = 1285) and 52.2% girls (*n* = 1404), with at least 127 to 265 subjects (mean 184.8) per age and per gender; falling within the criteria of a representative sample. Further, given the known impact of sociodemographic factors on nutritional status in children, a strength of this study was that sampling was stratified to assess a wide range of existing social strata, including primary and high schools, both public and private ones. While prevalence data obtained in this study cannot absolutely be generalized to every urban city in Cameroon, as notable social differences exists, this data from the largest Cameroon cities is representative of the Cameroon urban setting and can be compared to prevalence data recorded in other leading urban cities in other countries.

The results of this study showed that, in general, regardless of the reference parameter used, overweight and obesity affect an important percentage of children and adolescents in an area and a continent that has typically not been associated with this problem. To our knowledge, this is the first study to report the prevalence of overweight, obesity, and leanness in Cameroon children and adolescents with such a large sample and specifically the comparison of data to several existing indices. We noted major differences between our Database analysis and published reports, according to the reference used [25, 26]. IOTF showed 12.5% overweight including obesity, while similar cut-offs derived from our Database yielded 15.0%. French cut-offs showed the lowest percentage (10.9%), while US references showed intermediate values of 11.8% and 13.2%, respectively, for Must et al. and CDC references. Finally, WHO 2007 showed the highest percentage (17.8%). Interestingly, the greatest differences were accounted for by obesity alone, which reached 7.2% according to WHO 2007 compared to only 1.9% according to IOTF, although the prevalence of overweight alone was apparently the same (10.6% versus 10.5%, resp.). Similar trends were previously observed in a sample of Brazilian children, where evaluation of relative obesity/overweight differed according to the reference database used [22]. Based on this variation, which was dependent on the reference database used and the necessity and usefulness for clinicians evaluating weight and height for age to have population specific information, it is doubly important to establish a national Cameroon reference in order to evaluate ongoing changes in population parameters and weight change management by clinicians, especially in children during their developmental stages. Our current study provides a basis for this.

Gender showed a high impact on the prevalence of overweight and obesity as prevalence in girls (IOTF, 16.24%) was about twice that in boys (IOTF, 8.40%). The same trend remained, irrespective of the reference parameter used. Similar findings were reported in the Cameroonian adult population [3]. A similar wide variation in gender effect was noted in a South African 2001 to 2004 study [6], where black boys showed lower obesity (2.1%) as compared to black girls (4.7%) [27]. By contrast, countries such as China and India showed the inverse profile, with boys being heavier than girls [28], while in Westernized countries including the US and France, no such differences were noted [5, 26]. Based on the IOTF reference, 18.1% of US boys fall above the cut-off for overweight, with 4.7% for Brazilian boys [8]. Here we observed 8.4% for boys, according to the same cut-offs. For girls, 16.5% in the US were overweight, with 15.2% in Brazil [8], and in our Database, 16.2% of Cameroonian girls. The same feature was noted with other reference indices, with boys being less overweight or obese. These findings were supported by the overall significantly higher anthropometric variables measured (height, weight, and BMI) in girls as compared to boys. Clearly ethnic background and social environment impact on the prevalence of overweight and obesity in a gender-specific manner, again highlighting the importance of evaluating the Cameroon population based on a Cameroon-derived database.

Previous studies have noted a progressive reduction in thinness in developing countries facing social changes, while overweight and obesity are increasing [5, 6]. In the present study, we report that thinness corresponding to the adult cutoff of 18.5 kg/m^2^ was 9.7% in girls and 9.4% in boys; the uses of BMI cutoff corresponding to 18.5 kg/m^2^ at 18 years rather than BMI of 17 kg/m^2^ at 18 years suggested earlier [9] could explain the relative high prevalence of thinness. These values of thinness are still higher than reported data in developed countries such as the United States at 3.3% [5], France at 6% [26], and in a transitional country such as Brazil at 8.8% [5]. Although the prevalence of thinness is similar to data published previously in 12-to-15-year-old Cameroon adolescents with 10% of thinness [29] or in South Africa, with 10% [30], they point towards a shift in the population towards increasing overweight and obesity. When taking into consideration combined underweight, wasting, and stunting groups, the gender differences remained significant, as more boys were underweight and stunted while more girls were within the wasting group. These results are consistent with the existing sex dichotomy reported in the adult population of Cameroon with 9.8% men and 7.0% women, respectively, under a BMI of 20 kg/m^2^ [3].

## 5. Conclusion

In conclusion, to our knowledge, this study is among the first in Cameroon pointing out the prevalence of grades of nutritional status in children and adolescents at the urban level. As reference indices and cut-offs are often population-specific and sensitive and obesity/overweight varies widely worldwide, this highlights the importance of establishing a Cameroon-based reference. This study could then serve as a baseline and contribute to ongoing evaluation of the adverse effects of nutrition in transition. The important findings presented here are (i) a relatively high prevalence of overweight and obesity, compared to what was expected in this population, especially in girls, and (ii) yet at the same time, maintenance of thinness which is more prevalent in boys. Further studies are needed to follow the influence of socioeconomic environment on nutritional status grades in the context of economic growth.

## Figures and Tables

**Table:** (a)

Age (years)	8	9	10	11	12	13	14	15
*N*	106	125	130	163	194	224	203	140
Height (cm)	127.3 (0.64)	132.6 (0.76)	136.9 (0.75)	142.1 (0.62)	148.2 (0.63)	153.1 (0.69)	158.3 (0.67)	163.7 (0.76)
Weight (kg)	25.7 (0.38)	29.1 (0.59)	31.6 (0.63)	36.0 (0.50)	40.3 (0.53)	44.5 (0.65)	47.7 (0.56)	53.1 (0.82)
BMI (kg/m²)	15.8 (0.20)	16.3 (0.20)	16.7 (0.21)	17.7 (0.17)	18.2 (0.17)	18.8 (0.21)	18.9 (0.17)	19.7 (0.20)

*N*: number, BMI: body mass index, and values are given as mean (standard error of the mean).

**Table:** (b)

Age (years)	8	9	10	11	12	13	14	15
*N*	118	133	139	173	238	229	195	179
Height (cm)	128.6 (0.64)	132.3 (0.59)	138.1 (0.67)	146.7 (0.62)	152.7 (0.54)	156.4 (0.42)	159.3 (0.45)	160.3 (0.46)
Weight (kg)	27.4 (0.58)	28.5 (0.51)	32.9 (0.57)	40.2 (0.68)	45.1 (0.58)	49.3 (0.62)	52.6 (0.67)	55.6 (0.71)
BMI (kg/m²)	16.4 (0.23)	16.1 (0.20)	17.1 (0.18)	18.5 (0.24)	19.2 (0.20)	20.1 (0.22)	20.6 (0.23)	21.6 (0.24)

*N*: number, BMI: body mass index, and values are given as mean (standard error of the mean).

**Table 2:** Age specific *L*, *M*, *S*, and *z*-scores values for BMI (kg/m^2^) from Cameroon study population using LMS method, with additional *z*-scores and percentiles: Boys.

Age (years)	*n*	*L*	*M*	*S*	*z*-scores						85th	95th	97th
					−2	−1.33	−0.67	0.67	1.33	2	1.03	1.64	1.88
8	106	2.31	15.8	0.13	10.5	12.6	14.3	17.1	18.3	19.4	17.8	18.9	19.2
9	125	1.71	15.8	0.14	10.8	12.6	14.3	17.3	18.6	19.9	18.0	19.2	19.7
10	130	2.08	16.4	0.14	10.5	12.8	14.7	17.9	19.3	20.5	18.7	19.9	20.3
11	163	1.25	17.3	0.12	12.7	14.3	15.8	18.8	20.2	21.6	19.5	20.8	21.3
12	194	1.26	18	0.13	13.0	14.7	16.4	19.6	21.1	22.6	20.4	21.8	22.3
13	224	2.43	18.3	0.16	9.14	13.2	16.0	20.2	21.9	23.4	21.1	22.6	23.1
14	203	1.98	18.9	0.12	13.1	15.3	17.1	20.4	21.9	23.2	21.2	22.5	23.0
15	140	0.49	19.5	0.12	14.9	16.4	17.9	21.1	22.9	24.7	22.1	23.7	24.3

*n*: sample size, *L*: skewness, *M*: mean, and *S*: coefficient of variation. −2, −1.33, −0.67, 0.67, 1.33, and 2 are *z*-scores used to calculate 2nd, 9th, 25th, 75th, 91st, and 98th BMI percentiles, respectively.

**Table 3:** Age specific *L*, *M*, *S*, and *z*-scores values for BMI (kg/m^2^) from Cameroon study population using LMS method, with additional *z*-scores and percentiles: Girls.

Age (years)	*n*	*L*	*M*	*S*	*z*-scores						85th	95th	97th
					−2	−1.33	−0.67	0.67	1.33	2	1.03	1.64	1.88
8	118	1.14	15.9	0.15	10.9	12.6	14.3	17.6	19.2	20.8	18.5	19.9	20.5
9	133	1.09	15.9	0.14	11.2	12.8	14.4	17.5	19.0	20.5	18.3	19.7	20.2
10	139	0.59	16.9	0.12	12.8	14.1	15.5	18.4	19.9	21.5	19.2	20.7	21.2
11	173	1.85	17.7	0.17	10.2	13.1	15.6	19.7	21.5	23.2	20.7	22.3	22.9
12	238	1.18	18.7	0.16	12.4	14.6	16.6	20.7	22.6	24.6	21.8	23.6	24.2
13	229	1.02	19.5	0.16	12.9	15.1	17.3	21.7	23.9	26.1	22.9	24.9	25.7
14	195	1.08	20.3	0.15	13.8	16.0	18.1	22.4	24.5	26.6	23.6	25.5	26.2
15	179	0.74	21.3	0.14	15.2	17.2	19.2	23.4	25.6	27.9	24.6	26.7	27.5

*n*: sample size, *L*: skewness, *M*: mean, and *S*: coefficient of variation. −2, −1.33, −0.67, 0.67, 1.33, and 2 are *z*-scores used to calculate 2nd, 9th, 25th, 75th, 91st, and 98th BMI percentiles, respectively.

**Table 4:** Age- and gender-specific BMI cutoffs for the classification of nutritional status, obtained by using LMS method applied to our Cameroon database.

Age (years)	Thinness III		Thinness II		Thinness I		Overweight		Obesity	
	Boys	Girls	Boys	Girls	Boys	Girls	Boys	Girls	Boys	Girls
8	9.9	10.7	11.4	11.5	13.3	12.8	18.6	17.9	21.3	21.6
9	10.3	11.1	11.6	11.9	13.3	13.0	18.9	17.8	22.2	21.3
10	9.9	12.6	11.5	13.3	13.6	14.3	19.5	18.7	22.7	22.3
11	12.3	9.9	13.4	11.4	14.9	13.5	20.5	20.1	24.1	24.0
12	12.6	12.2	13.8	13.2	15.4	14.8	21.4	21.1	25.4	25.6
13	7.5	12.7	11.1	13.8	14.4	15.4	22.2	22.2	25.9	27.2
14	12.5	13.5	14.0	14.6	16.0	16.2	22.2	22.8	25.6	27.6
15	14.6	15.0	15.5	15.9	16.9	17.4	23.2	23.9	28.3	29.1

Thinness III, Thinness II, Thinness I, overweight, and obesity correspond, respectively, to BMI values of 16, 17, 18.5, 25, and 30 kg/m² at 18 years old.

**Table 5:** Prevalence (%) of overweight and obesity in the study population according to different references.

References	Boys (*n* = 1285)		Girls (*n* = 1404)		Boys + girls (*n* = 2689)		Chi-squared test
	Overweight^†^	obese	Overweight^†^	obese	Overweight^†^	obese	*P* value
*Method 1*							
Database lms	6.4	2.2	17.2	3.8	12.0	3.0	—
IOTF [8]	7.0	1.4	13.8	2.4	10.5	1.9	<0.001
*Method 2*							
Database 85th	6.6	7.0	7.9	8.2	7.3	7.6	—
WHO 2007 [21]	8.2	5.5	12.9	8.6	10.7	7.2	<0.001
Must et al. [18]	6.4	2.4	10.7	3.7	8.6	3.1	<0.001
CDC [19, 20]	6.4	3.0	12.4	4.1	9.5	3.6	<0.001
*Method 3*							
Database 97th*	5.7	—	6.2	—	5.9	—	—
French 97th* [11]	6.8	—	14.7	—	10.9	—	<0.001

^†^Overweight excluding obesity, *overweight including obesity, International Obesity Task force (IOTF), World Health Organization (WHO), and Center of disease Control (CDC), difference between database and corresponding references was calculated by Chi-squared test, and *P* < 0.05 was set at significant.

**Table 6:** Prevalence of undernutrition defined as underweight (low weight for age), stunting (low height for age), wasting (low weight for height) and thinness (low BMI for age) based on several references used: WHO 2007, CDC, Cole et al. 2007, and our Database.

Reference	Status	Range*	Boys: *n*/*N* (%)		Girls: *n*/*N* (%)		Boys + girls	
WHO 2007 [21]	Underweight	<10 yrs	20/361	(5.5)	19/390	(4.8)	39/751	(5.2)
	Stunting	8/15 yrs	119/1285	(9.2)	34/1404	(2.4)	153/2689	(5.7)
	Wasting	<163 cm	21/1256	(1.6)	36/1245	(2.9)	57/2501	(2.2)
	Wasting I	<163 cm	18/1256	(1.4)	31/1245	(2.5)	49/2501	(1.9)
	Wasting II	<163 cm	3/1256	(0.2)	5/1245	(0.4)	8/2501	(0.3)

CDC [19, 20]	Underweight	8/15 yrs	77/1285	(5.9)	38/1404	(2.7)	115/2689	(4.2)
	Stunting	8/15 yrs	118/1285	(9.2)	39/1404	(2.7)	157/2689	(5.8)
	Wasting	<121 cm		—	2/30	(6.6)	2/71	(2.8)
	Wasting I	<121 cm		—		—		—
	Wasting II	<121 cm		—		—		—

Cole et al. 2007 [9]	Thin	8/15 yrs	121/1285	(9.4)	136/1404	(9.7)	257/2689	(9.5)
	Thin I	8/15 yrs	285/1285	(7.3)	96/1404	(6.8)	190/2689	(7.1)
	Thin II	8/15 yrs	18/1285	(1.4)	20/1404	(1.4)	38/2689	(1.4)
	Thin III	8/15 yrs	9/1285	(0.7)	20/1404	(1.4)	29/2689	(1.1)

Our Database	Thin	8/15 yrs	71/1285	(5.5)	70/1285	(4.9)	141/2689	(5.2)
	Thin I	8/15 yrs	61/1285	(4.7)	54/1285	(3.8)	115/2689	(4.2)
	Thin II	8/15 yrs	8/1285	(0.6)	13/1285	(0.9)	21/2689	(0.8)
	Thin III	8/15 yrs	2/1285	(0.1)	3/1285	(0.2)	5/2689	(0.2)

*n*: outcome, *N*: total exposed, and *range defines the limit or the subset to which the cutoffs were valid or available in the literature. World Health Organization (WHO) and Center of disease Control (CDC).
